# “A Great Synthesis of Labor, Light, and Movement”

**DOI:** 10.3201/eid2808.AC2808

**Published:** 2022-08

**Authors:** Byron Breedlove

**Affiliations:** Centers for Disease Control and Prevention, Atlanta, Georgia, USA

**Keywords:** art science connection, emerging infectious diseases, art and medicine, about the cover, a great synthesis of labor, light, and movement, elasticity, Umberto Boccioni, Italian futurism, cubism, World War I, public health, 1918‒1919 influenza pandemic, influenza, influenza virus, H1N1 virus, viruses, One Health, zoonoses

**Figure:**
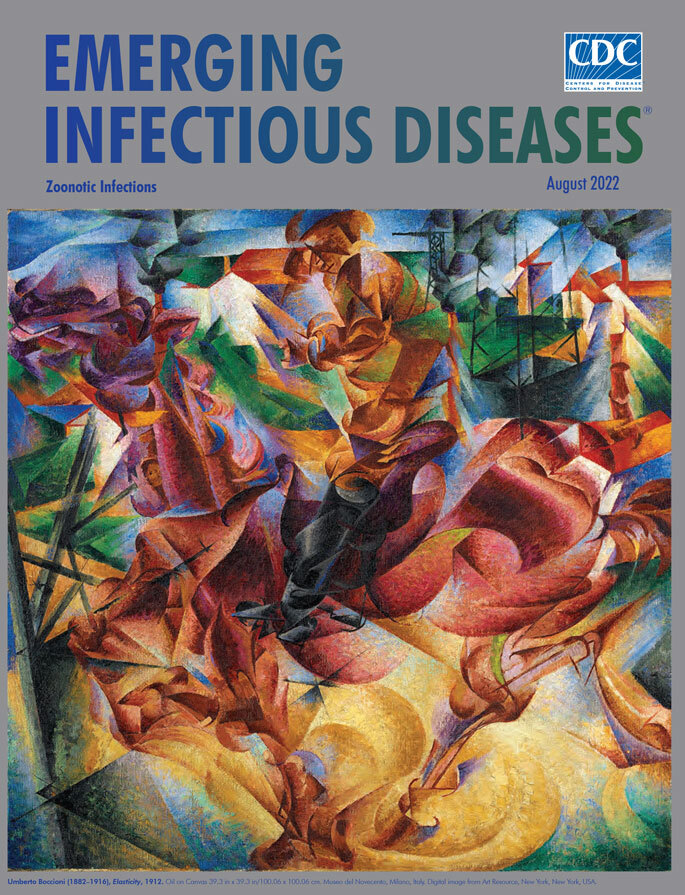
**Umberto Boccioni (1882–1916), *Elasticity*, 1912.** Oil on Canvas 39.3 in x 39.3 in/100.06 cm × 100.06 cm. Museo del Novecento, Milano, Italy. Digital image from Art Resource, New York, New York, USA.

“Let us not offend Boccioni with a funeral eulogy,” stated Italian writer Filippo Tommaso Marinetti, founder of the early 20th Century avant-garde Italian Futurist movement, at a retrospective exhibition for Italian painter and sculptor Umberto Boccioni. Despite a life cut short, Boccioni was one of the best known and most influential artists associated with early manifestations of Italian Futurism. Boccioni was born in the coastal Italy city of Reggio Calabria, but his family moved frequently during his childhood. After attending technical college in Catania, Sicily, in 1899 he studied drawing and painting in Rome. In 1906, Boccioni visited Paris to expand his artistic perspectives and then spent a portion of 1907 in Venice learning printmaking before relocating to Milan, where he met Marinetti. 

Art historian Rosalind McKever noted, “In February 1909, Marinetti published his famous manifesto in the French newspaper *Le Figaro*, demanding that Italian culture stop looking backward and embrace modernity.” Boccioni and his peers drafted a pair of related manifestos calling on other artists to discard traditional motifs and conventions and to find inspiration from science, technology, and modern urban life. In Boccioni’s words, this artistic response would be “a great synthesis of labor, light, and movement.” A 1911 trip to Paris opened his eyes to the artistic style known as Cubism, and as McKever wrote, “Boccioni integrates the Cubist use of collage-like numerals and straight lines that divide spatial planes into his more expressionistic style… the dissected planes implied dynamism and the straight lines became ‘force-lines’ showing motion.” 

Boccioni’s painting *Elasticity*, this month’s cover image, fuses stylistic elements from Cubism with themes from Futurism. Its fractured, chaotic elements also may serve as a visual metaphor calling for collective, focused public health action in response to an array of emerging zoonotic diseases. 

Art historian Kate Bryan wrote, “Boccioni’s subject matter was not still life, as had been largely the case with Cubism, to which the movement owed a clear stylistic debt, but rather the more impossible endeavour of depicting objects in motion.” Horse and rider frantically gallop across a landscape that is the antithesis of a bucolic, pastoral setting. Jutting electrical towers, rendered as fractured angular black lines and factory chimneys belching smoke, reveal a setting reshaped by human hands. Angular forms and planes representing glimpses of sky and artifacts of industry recede from the horse and rider they surround. The distorted, dynamic image of the horse with its flaring nostrils, sweeping forelock, and rippling muscles, and its forward-leaning rider, wearing a brimmed hat and black boots, trampling across a dusty road, transfixes the viewer’s attention toward the center of the painting. Artist and writer Marcus Bunyan commented that in *Elasticity*, Boccioni depicted “the pure energy of a horse, captured with intense chromaticism.” 

As is already clear, Boccioni did not have a long career or life. In 1915, he volunteered for military service in the first World War. Although not a casualty of combat, Boccioni succumbed to severe injuries after being thrown from his horse startled by a passing lorry in August 1916. McKever noted, “Throughout his career Boccioni had painted horses and it is a cruel irony that, having named his steed Vermiglia after the flaming red beast in the centre of *The City Rises* (1910), his fall would imitate the scene of what has become his most famous painting.” Many of Boccioni’s generation also died during World War I, including his Futurist colleague Antonio Sant’Elia and artists Henri Gaudier-Brzeska and Franz Marc. 

Overlapping that global conflict was the 1918 influenza pandemic, caused by what would later be categorized as an H1N1 virus with genes of avian origin, which eventually infected one third of the world’s population. Of that pandemic, Taubenberger and Morens wrote, “Many questions about its origins, its unusual epidemiologic features, and the basis of its pathogenicity remain unanswered.” Among the human-mediated factors that drove the high rates of death and illness associated with that pandemic were wartime conditions, marshalling of military operations, mass transportation by ship and rail, and growing urbanization, which would have been celebrated by Futurists as transformative forces. 

More than a century later, the web of interrelated factors contributing to the emergence and reemergence of zoonotic infectious diseases has become more complicated and intertwined. Efforts to mitigate this complex global problem, if fragmented like the myriad shapes and shards in Boccioni’s *Elasticity*, are unlikely to succeed. As Ghai et al. noted, “Effectively preventing and controlling zoonotic diseases requires a One Health approach that involves collaboration across human health, animal health, and environmental sectors, as well as other partners. This framework provides a structure for using a One Health approach in zoonotic disease programs and can help build capacity for preventing and controlling zoonotic diseases at the local, sub-national, national, regional, or international level.” The urgency encapsulated in Boccioni’s notion of “a great synthesis of labor, light, and movement” is needed to drive this unified public health framework to prevent, prepare for, and respond to emerging zoonotic diseases.

## References

[1] Beard L, Butler A, Cleave CV, Fortenberry D, Stirling S. The art book. London: Phaidon Press; 2014.

[2] Bryan K. Bright stars: great artists who died too young. London: Francis Lincoln; 2021.

[3] Bunyan M. Art Blart. Umberto Boccioni [cited 2022 Jun 29]. https://artblart.com/tag/umberto-boccioni-elasticity

[4] Centers for Disease Control and Prevention. History of 1918 flu pandemic [cited 2022 Jun 23]. https://www.cdc.gov/flu/pandemic-resources/1918-commemoration/1918-pandemic-history.htm

[5] Centers for Disease Control and Prevention. Zoonotic diseases [cited 2022 Jun 14]. https://www.cdc.gov/onehealth/zoonotic-diseases.html

[6] Coen E. Umberto Boccioni: a retrospective. New York: Metropolitan Museum of Art; 1988.

[7] Ghai RR, Wallace RM, Kile JC, Shoemaker TR, Vieira AR, Negron ME, et al. A generalizable one health framework for the control of zoonotic diseases. Sci Rep. 2022;12:8588. 10.1038/s41598-022-12619-1 35597789 PMC9124177

[8] McKever R. Harnessing the future: the art of Umberto Boccioni [cited 2022 Jun 4]. https://www.apollo-magazine.com/harnessing-the-future-the-art-of-umberto-boccioni

[9] McKever R. Umberto Boccioni (1882–1916) [cited 2022 Jun 14]. https://www.metmuseum.org/toah/hd/umbo/hd_umbo.htm

[10] Short KR, Kedzierska K, van de Sandt CE. Back to the future: lessons learned from the 1918 influenza pandemic. Front Cell Infect Microbiol. 2018;8:343. 10.3389/fcimb.2018.00343 30349811 PMC6187080

[11] Taubenberger JK, Morens DM. 1918 Influenza: the mother of all pandemics. Emerg Infect Dis. 2006;12:15–22. 10.3201/eid1209.05-0979 16494711 PMC3291398

